# Validation and minimally important difference of the Child-OIDP in a socioeconomically diverse sample of Indian adolescents

**DOI:** 10.1186/s12955-022-01949-3

**Published:** 2022-04-27

**Authors:** Manu Raj Mathur, Deepti Nagrath, Huda Yusuf, Vijay Kumar Mishra, Georgios Tsakos

**Affiliations:** 1 Public Health Foundation of India, Plot No. 47 Sector 44, Institutional Area, Gurgaon, 122002 Haryana India; 2 University of Liverpool, Liverpool, L69 3BX UK; 3 Consultant in Dental Public Health at Public Health England, Training Programme Director for Dental Public Health for London and KSS- Health Education England, Centre for Dental Public Health and Primary Care, Institute of Dentistry, Barts and The London School of Medicine and Dentistry, Queen Mary University of London, London, UK; 4 Department of Epidemiology and Public Health, University College London, Gower Street, London, WC1E 6BT UK

**Keywords:** Oral health related quality of life (OHRQoL), India, Oral impacts on daily performances (OIDP), Minimally important difference, Psychometrics, Validity, Adolescents, Slums

## Abstract

**Introduction:**

While different measures have been validated and used to assess the oral health related quality of life (OHRQoL) of children and adolescents, no previous study has tested the psychometric performance of OHRQoL amongst the most marginalized adolescents, living in extremely deprived neighbourhoods like urban slums and resettlement areas in modern cities. Our study assessed the internal consistency reliability, construct validity and Minimally Important Difference (MID) of the Child-OIDP in a sample of adolescents aged 12–15 years reporting oral health problems that lived in three different types (including two extremely vulnerable) of neighbourhoods (urban slums, resettlement colonies, and middle and upper middle-class neighbourhoods) in the National Capital Territory of Delhi.

**Methods:**

We conducted data analysis on a cross-sectional study, comprising of 840 adolescents. The Child-OIDP was used as a measure of OHRQoL. Internal consistency reliability was tested using the standardized Cronbach’s Alpha Coefficient. The Child-OIDP was also tested for content and construct validity (the latter through the median test), while a distribution-based approach was used to identify the MID.

**Results:**

The Indian Child-OIDP showed good internal consistency, as the Cronbach’s alpha coefficient was 0.77. Inter-item correlation coefficients among the items ranged from 0.13 to 0.50, with the mean inter-item correlation being 0.30. The corrected item-total correlations ranged from 0.30 (social contact) to 0.54 (speaking). For construct validity, the Child-OIDP extent was significantly associated with three subjective oral and general health variables in the expected direction. The calculated effect sizes for these differences indicated that they were moderate (0.50–0.79). We also calculated the standard error of measurement (SEM) of Child-OIDP extent as 0.75.

**Conclusion:**

This study demonstrated that the Indian Child-OIDP is a reliable and valid measure for the assessment of the oral health related quality of life among Indian adolescents especially from marginalised and socioeconomically vulnerable groups. This is an essential step towards assessing oral health and evaluating oral health promotion interventions in those populations and settings.

## Introduction

Comprehensive assessment of a person’s experience with a condition helps in identifying specific treatment needs and improving patient care, with the overall aim to improve the quality of life of individuals and populations [1]. This recognises health as a multidimensional concept that extends beyond simply the presence or absence of diseases. Oral Health Related Quality of Life (OHRQoL), is defined as *“a multidimensional construct that reflects (among other things) people's comfort when eating, sleeping, and engaging in social interaction; their self-esteem; and their satisfaction with respect to their oral health* [2]*.* OHRQoL is often used to complement clinical indicators as it takes social, psychological, physical and functional outcomes into account [2]. The concept of OHRQoL is important for designing health policies, allocating health resources, and planning disease prevention programs. It encompasses the functional, social, and psychological aspects of oral health [3]. Measuring oral impacts in children and adolescents is important for researchers and health policy makers, as it can facilitate the comprehensive assessment of oral health needs, prioritising care within limited resources and evaluating treatment outcomes.

Various measures have been devised to assess OHRQoL among children. The Child-Oral Impacts on Daily Performances (Child-OIDP) [4] is one of the widely used measures globally. The Child-OIDP quantifies the impact of oral conditions on daily activities of children, such as eating, speaking, cleaning teeth, smiling, emotional stability, relaxing, doing schoolwork/homework, and social contact [4]. It has been used in various countries, cultural settings, and age groups, with a number of studies having evaluated its reliability and validity [3–10]. Studies have also tested the Child-OIDP on young adolescents [11–13] which are the formative years in the life cycle of an individual. Adolescents start gaining independence and become more aware of their physical appearance, social environment, and perceived needs. They start making their own behavioural and dietary choices as well as being concerned with their appearance. It is therefore important to assess OHRQoL during this period. Many previous studies from different countries have assessed OHRQoL in school children but have not included the ones from extremely vulnerable socio-economic groups [10, 13–17] including those from the Indian subcontinent [18, 19].

Furthermore, while studies have focused on the assessment of the psychometric properties of OHRQoL measures, they have by large neglected to address the issue of their interpretability [20]. This is achieved through assessing the Minimal Important Difference (MID), defined as “*The smallest difference in score in the outcome of interest that informed patients or informed proxies perceive as important, either beneficial or harmful, and that would lead the patient or clinician to consider a change in the patient’s management”* [21]. The concept gained its importance in determining whether the observed change is meaningful [22]. To the best of our knowledge, none of the aforementioned studies has tested the reliability and Minimal Important Difference (MID) amongst poorest and most marginalized adolescents especially those with oral health problems, living in extremely deprived neighbourhoods like urban slums and resettlement areas in modern cities, where the living conditions are indicative of excessive deprivation, reflecting the cliff-edge of inequalities, without even the basic facilities to maintain good health. An appropriate measure of OHRQoL in those populations and settings is an essential step towards assessing oral health and evaluating oral health promotion interventions.

We therefore, undertook this study in order to assess the internal consistency reliability, construct validity and MID of the Child-OIDP amongst a sample of adolescents reporting oral health problems that lived in three different types of neighbourhoods (urban slums, resettlement colonies, and middle and upper middle-class neighbourhoods) in the National Capital Territory of Delhi.

## Methods

### Study population and sample

We undertook a cross sectional study on adolescents aged 12–15 years, living in the National Capital Territory (NCT) of Delhi, and selected adolescents residing in areas representative of three different socioeconomic groups: urban slums, resettlement colonies, and middle-class neighbourhoods.

According to the Census of India in 2001 [23], urban slum areas lack security of environment, livelihood, amenities and tenure. These slums have been characterized by harsh physical and social environmental conditions like poor housing, insecurity of tenure, poor access to safe drinking water, sanitation and severe overcrowding [24]. Resettlement colonies are located adjacent to a slum. The Government provides more infrastructure (e.g., water and electricity) in these colonies, however living conditions can still be very difficult. To summarize, a resettlement colony is much larger than a slum, with thousands of households organized into blocks.

Slums and resettlement colonies were recruited from a list of registered resettlement colonies and urban slums in Delhi. The inclusion criteria were: (a) colonies must be within a radius of 25 km from the research office, (b) both slum and resettlement colony should present together as a cluster, (c) colonies should have more than 500 households in both the components of the cluster (slums and resettlement colonies), and (d) a known non-governmental organization should be working in the community, willing to participate in the research. Fourteen colonies and slums were found to be eligible. In order to assess the variation in their population features, demographic data from two blocks in each of these slums and resettlement colonies were collected. All the slums and resettlement colonies were found to be demographically comparable (in terms of ethnicity, religion, language, number of households, population per block, and school going/non-school going children per family). For the adolescents from middle class neighbourhoods, private schools which have English language as medium of education and charge higher fees (‘English Medium Schools’) were selected. Multi-stage random sampling technique was used to select the study sample. Four slums and resettlement colonies were randomly selected from the 14 identified colonies and four English medium private schools were randomly selected from a sampling frame of 48 eligible schools.

### Recruitment

The parents of adolescents in urban slums and resettlement colonies were approached with the help of local NGO representatives working actively in that area and were introduced to the study through a written information sheet. An informed consent form was given to the participants and interviews were conducted once the parents signed the consent form. For parents who are unable to read and write, the interviewer/field worker explained the study, including benefits and possible risks in a simple and easy to understand language. Thumb impression of the parents on the consent form was taken if they were illiterate after fully explaining the study. For obtaining the sample of children from middle and upper middle-class homes, the relevant school authorities were contacted. The selected children were then given an information sheet providing details of the study and consent form, to be signed by their parents. Once parental consent was received, the children were also asked to provide their consent to be a part of the study. Adolescents were recruited only when both the parents and the adolescent had signed consent forms.

### Data used

We used relevant data from a large sample of adolescents residing in urban slums, resettlement colonies, and middle-class neighbourhoods in NCT Delhi. Overall, 1,600 adolescents were contacted and 1,386 agreed to participate (response rate: 86.6%). Of them, 840 participants reported “Yes” to the question *“Do you have any of these problems (had toothache or sensitive teeth, had bleeding or swollen gums, been aware of decay in your teeth or a broken adult tooth, had ulcers or a loose baby tooth, had a problem because of tooth colour, shape, size or position) in the last 3 months”* (Appendix). These 840 participants with self-reported oral health problems constitute the analytical sample for this study as the Child-OIDP questions (*“Have any of these problems with your teeth and mouth led to difficulties with Eating, Speaking, Cleaning your mouth, Relaxing (including sleeping),Your feelings (for example being more impatient, irritable, easily upset), Smiling or laughing, Doing your schoolwork, Mixing with friends and other people”)* were addressed only to them.

### Study measures

Data for this analysis were collected through an interviewer-administered questionnaire. The questionnaire measured material resources, neighbourhood social capital, social support, health-related behaviours (alcohol and tobacco use, diet, frequency of tooth brushing), and key health and sociodemographic variables.

The questions on health behaviours were derived from the WHO HBSC survey questionnaire and were aimed to assess habits like frequency of brushing, tobacco and alcohol consumption, dietary pattern and involvement in violent activity. Self-rated oral health of adolescents was measured by asking respondents as to how do they describe the overall condition of their teeth and gums [25].

The questionnaire included pre-existing questions and scales which were checked for reliability and validity in the study population during a pilot study to assess material deprivation in India [26]. The response from the students was very positive and we achieved a response rate of 86.6%.

Translation of the questionnaire in Hindi and back translation to English was independently done by two people who were proficient in both languages (English and Hindi). It was also back translated in order to ensure that the true meaning of the questions was not lost in the Hindi version. Translation of questionnaire in Hindi was done keeping in mind the sensitivity to the local culture.

### Variables

The Child–OIDP questionnaire was used as the measure of OHRQoL in this study. It assessed oral impacts on the following daily performances: eating, speaking, cleaning teeth, smiling, emotional stability, relaxing (including sleeping), doing schoolwork/homework, and social contact.

The Child-OIDP extent was created by counting the number of oral impacts on the aforementioned daily performances and ranged between 0 and 8, with every unit representing one more oral impact reported. The explanatory variables included in the study were ‘Age’, ‘Gender’, ‘Educational level of adolescent’, ‘area of residence’, ‘wealth index’, ‘bothered by oral health problem ‘, self-rated oral health’ and ‘self-rated general health’.

The wealth of the adolescent’s households was assessed by asking them questions about various material assets (television, car, electricity at home, bicycle, built-in kitchen sink, hot running water, washing machine, dishwasher, refrigerator, domestic help, mobile/cellular phone, bullock cart, computer, stereo system, livestock, internet access, motorbike and a second home). The responses on these households’ assets were used to create the variable “wealth index” using Principal Component Analysis (PCA). The variable ‘wealth index’ which was used to understand the socio-economic status (SES) of adolescents, was categorized as ‘poor’, middle’ and ‘rich’ [27]. The perception on difficulty due to oral problems was assessed by the question- “*To what extent have you been bothered by the problems of your mouth and teeth?”*, and its related variable ‘bothered by oral health problems ‘was generated on dichotomizing the responses into ‘yes = 1’ and ‘no = 0’. Similarly, the perception on oral health and general health were assessed by the questions *“How would you describe the overall condition of your teeth, denture and gums?”* and *“How would you describe your overall health?”*. We also dichotomised the responses of these variables (self-rated oral health and self-rated general health) into ‘good = 1’ and ‘bad = 0’ for analysis purposes.

### Data analysis

The internal consistency reliability was tested by using the standardised Cronbach’s alpha coefficient, item–total and inter–item correlations.

As mentioned earlier, of the 1,386 adolescent children 840 adolescents reported experiencing an oral health problem (toothache or sensitive teeth, bleeding or swollen gums, decayed or broken tooth, ulcers, tooth discolouration) and were subsequently asked the Child-OIDP questions. These adolescents comprise the analytical sample for this study. Descriptive analysis included percentage distribution of categorical variables. Bivariate analysis using Chi-squared test at 5% significance level (two-tailed) was used to understand the association between outcome and demographic (age, gender), socio-economic (education, place of residence, wealth index) and health variables (self-rated oral health, self-rated general health, bothered by oral health problem). The Child-OIDP extent score varied from 0 to 8. We further dichotomised the Child-OIDP extent into those with no oral impact (Child-OIDP = 0) and those with at least one oral impact (Child-OIDP = 1) to understand the association between prevalence of oral impacts and other selected variables.

### Content and Face validity

Content validity of the Indian version of the Child-OIDP was established by seeking feedback from subject matter experts in oral health, non-communicable diseases and social determinants of health. A pilot study was also undertaken on an independent sample of 50 adolescents to further assess the acceptability and confirm the appropriateness of the layout, translation and sequence of questions.

During the pilot study, the respondents were asked to provide input on the way questions were asked, their perceived difficulty in understanding a particular question, the sequencing of questions and whether anything important has been missed. This helped us to establish the face validity of the Indian Child-OIDP.

### Construct validity

The construct validity was assessed by looking at the associations between the Child-OIDP (extent) and three variables (‘self-rated oral health’, ‘self-rated general health’ and ‘bothered by oral health problems’) through a non-parametric test (median test) on the distribution of the OIDP extent.

### Minimally important difference (MID)

The study utilized the distribution-based approach to calculate the MID for the Child-OIDP extent. Distribution based MID methods, i.e., Effect Size (ES) and Standard Error of Measurement (SEM) were used due to the cross-sectional nature of data [22]. To estimate the ES and SEM, we dichotomized the variables ‘self-rated oral health’, ‘self-rated general health’ and ‘bothered by oral health problems”. ES is defined as the difference in mean scores between groups divided by the standard deviation of both groups. The magnitude of ES < 0.20 SD, 0.20–0.49SD, 0.50–0.79 and ≥ 0.80 SD is classified as negligible, small, moderate, and large, respectively. SEM was estimated by standard deviation multiplied by a square root of one minus the internal consistency of the Child-OIDP extent. All statistical analyses were performed using Stata software (version-14.0) [28].

## Results

The respondents as well as the interviewers were satisfied with the wording, sequencing and appropriateness of questions. The mean age of the adolescents who reported at least one oral symptom (N = 840) was 13.2 years. 62.5% children in the age-group 12–13 years experienced one or more oral impacts, followed by 47.2% in the 14–15 years age-group. 58% of girls and 54.5% of boys reported at least one oral impact on their daily life. 64.7% of illiterate children faced at least one oral impact, followed by those who completed primary (60.5%) or secondary education (60.3%), while 46.4% adolescents who completed higher education reported at least one oral health problem. Similarly, 60.3% of adolescents residing in slums experienced at least one oral impact, followed by those who were from resettlement colonies (56.9%), and adolescents living in upper/middle class (49.6%). Nearly 61% of adolescents belonging to poor socio-economic status experienced at least one oral impact, followed by 53.1% of adolescents who belonged to middle SES. The Child-OIDP was associated with socio-economic (except gender) and other factors (Table 1)**.**

**Table 1:** Prevalence of oral impacts among adolescents who reported oral symptoms (swollen gums, ulcers, decayed tooth, tooth ache, tooth discolouration), by background characteristics (n = 840)

Background characteristics	Oral impacts (Child-OIDP)		*P* value
	No n (%)	Yes n (%)	
Age, mean (SD)	13.5 (1.2)	13.2 (1.1)	
Age group			
12–13 years	182 (37.5)	304 (62.5)	< 0.001
14–15 years	187 (52.8)	167 (47.2)	
Gender			
Boys	211 (45.5)	252 (54.5)	0.287
Girls	158 (41.9)	219 (58.1)	
Education of adolescents			
Illiterate	12 (35.3)	22 (64.7)	0.002
Primary	32 (39.5)	49 (60.5)	
Secondary	182 (39.7)	276 (60.3)	
Higher	143 (53.6)	124 (46.4)	
Area of residence			
Middle/upper middle	116 (50.4)	114 (49.6)	0.045
Resettlement colonies	138 (43.1)	182 (56.9)	
Slums	115 (39.7)	175 (60.3)	
Wealth index			
Poor	137 (38.8)	216 (61.2)	0.038
Middle	97 (46.9)	110 (53.1)	
Rich	135 (48.2)	145 (51.8)	
Bothered by oral health problem			
No	173 (60.3)	114 (39.7)	< 0.001
Yes	196 (35.4)	357 (64.6)	
Self-rated oral health			
Bad	29 (19.9)	117 (80.1)	< 0.001
Good	340 (49.1)	353 (50.9)	
Self-rated general health			
Bad	10 (20.4)	39 (79.6)	0.001
Good	359 (45.4)	432 (54.6)	
Total	369 (43.9)	471 (56.1)	

Overall, 56.1% reported at least one oral impact on their daily life. The most prevalent oral impact was difficulty eating (40.6%), followed by difficulty cleaning teeth (32.5%) and speaking (13.3%). Adolescents also reported considerable prevalence in terms of feeling different such as being impatient, irritable or being easily upset (13.6%) due to their oral health. Difficulty relaxing (9.2%) and avoiding smiling or laughing without embarrassment (7.6%) were less prevalent, while difficulty with schoolwork (3.3%) and social contacts (3.7%) were the least prevalent oral impacts (Table 2).

**Table 2:** Prevalence of oral impacts on daily performances (Child-OIDP) among adolescents that reported at least one oral symptom (swollen gums, ulcers, decayed tooth, toothache, tooth discolouration) (n = 840)

Oral impacts on daily performances (Child-OIDP)	Impact, n (%)
Difficulty eating	341 (40.6)
Difficulty cleaning your mouth	273 (32.5)
Difficulty speaking	112 (13.3)
Avoiding smiling	64 (7.6)
Difficulty relaxing (including sleeping)	77 (9.2)
Felt different (for example being more impatient, irritable, easily upset)	114 (13.6)
Difficulty with school work	28 (3.3)
Difficulty with social contacts (mixing with friends and other people)	31 (3.7)
At least one oral impact	471 (56.1)

The Child-OIDP showed good internal consistency reliability. The inter-item correlation coefficients ranged from 0.13 (for the relationship between cleaning and social contact) to 0.50 (for the relationship between feelings of impatience, irritability or getting upset easily and relaxing), and the mean inter-item coefficient was 0.30 (Table 3).

**Table 3:** Reliability analysis: inter-item correlation for the Child-OIDP (n = 840)

	Eating	Speaking	Cleaning	Relaxing	Feeling	Smiling	School work	Social contact
Eating	1.00							
Speaking	0.39	1.00						
Cleaning	0.47	0.47	1.00					
Relaxing	0.31	0.34	0.28	1.00				
Feeling	0.30	0.27	0.29	0.50	1.00			
Smiling	0.25	0.39	0.24	0.45	0.39	1.00		
School work	0.15	0.20	0.13	0.23	0.33	0.40	1.00	
Social contact	0.16	0.17	0.14	0.31	0.24	0.27	0.26	1.00

Furthermore, the corrected item-total correlation ranged from 0.30 (social contact) to 0.54 (speaking) and the Cronbach’s alpha coefficient was 0.77 and was lower when any of the Child-OIDP items were deleted (Table 4).

**Table 4:** Internal consistency reliability of the Child-OIDP index among adolescents (n = 840)

Items	Corrected item-total correlation	Cronbach’s alpha if item deleted
Eating	0.50	0.71
Speaking	0.54	0.69
Cleaning	0.51	0.70
Relaxing	0.52	0.70
Feeling	0.49	0.71
Smiling	0.32	0.73
School work	0.49	0.70
Social contact	0.30	0.74

Standardised item alpha = 0.77

The results of median test on OIDP extent and three subjective oral and general health variables (‘self-rated oral health’, ‘self-rated general health’ and ‘bothered by oral health problem’) showed significant associations in the expected direction (higher prevalence of oral impacts for groups with worse perceptions about their oral and general health) and provided evidence for the construct validity of the Child-OIDP for this population. The respective effect sizes indicated moderate (0.50 to 0.79) differences in the prevalence of oral impacts between distinct groups of the aforementioned subjective variables. We also calculated the standard error of measurement (SEM) of OIDP extent to be 0.75 (Table 5).

**Table 5:** Minimally importance difference of Child-OIDP extent based on distribution approach (N = 840)

	n	Median	Effect size	[95% Conf. Interval]		*P* value
Self-rated oral health						
Bad	146	2.0	0.55	0.37	0.73	< 0.001
Good	693	1.0				
Self-rated general health						
Bad	49	1.0	0.68	0.39	0.97	< 0.001
Good	791	1.0				
Bothered by oral health problem						
No	287	0.0	0.52	0.66	0.37	0.019
Yes	553	1.0				

SEM = SD_oidp_ * $$\sqrt[2]{1 - r}$$

SD_oidp_ = 1.58, Standardised alpha(r) = 0.77

SEM = 1.58 * $$\sqrt[2]{1 - 0.77} = 0.75$$

## Discussion

Our study showed that the Child-OIDP has good internal consistency reliability and validity when applied among Indian adolescents with oral symptoms. This is the first study to test the psychometric performance of the Child-OIDP (or any other OHRQoL measure) among a sample that included adolescents living in extremely deprived neighbourhoods like urban slums and resettlement areas. Cross-cultural adaptation of a measure often requires a laborious process to make the adopted measure culturally relevant for the local population [29]. However, the participants of the present study were familiar with English as a second mother tongue thus providing an added advantage in establishing the content validity of the employed questionnaire. For construct validity, significant associations were found between the Child-OIDP extent and self-rated general health, self-rated oral health and perceived satisfaction with appearance of teeth, suggesting that those satisfied with their overall oral health, general health and appearance of teeth had fewer oral impacts and therefore better quality of life than the adolescents with worse ratings in these variables. The findings were similar to OIDP studies in other populations [30, 31]. The Child-OIDP has been tested both in developed and developing countries [32]. In terms of internal consistency reliability analysis, all corrected item-total correlations were positive and above the recommended level of 0.2 for including an item in a scale [33]. The lowest corrected item-total correlation was 0.30 for social contact and the highest was for the items on difficulty speaking and difficulty eating (0.54 and 0.50, respectively). In relation to inter-item correlations, all were positive. The Cronbach’s alpha coefficient was 0.77 and this value was lower when any item was deleted. This demonstrates excellent internal consistency reliability and is in line with findings from studies utilising the Child-OIDP in other countries [32, 34].

The most prevalent oral impact referred to difficulty eating, followed by difficulty cleaning, in line with findings from studies using the Child-OIDP in other countries [30, 32, 35]. The overall prevalence of oral impacts was 56.1%, also comparable with that observed among adolescents in a Tanzanian study, using a similar methodology [36]. However, it is much higher than studies conducted on Indian schoolchildren [37, 38].

Furthermore, we calculated the MID, thereby allowing for better interpretability of the differences in Child-OIDP extent scores. Using ES, we showed that there were significant and moderate differences in Child-OIDP between groups with different ratings of their oral and general health. Those with better self-ratings of oral and general health had also lower prevalence of oral impacts. This indicates the satisfactory performance of the Child-OIDP in terms of construct validity.

Moreover, it also shows that the differences in OHRQoL between those with different perceptions of general and oral health were moderate in magnitude. While most studies in the literature focus on statistical significance for such differences, using the MID can provide an estimate of whether these differences are meaningful from a clinical or public health perspective. We also used the SEM and calculated that a difference of up to 0.75 in the Child-OIDP extent is likely a result of measurement error; therefore, differences larger than 0.75 (e.g. differences of one more oral impact reported) would be meaningful and could be used as a yardstick to determine the relevance of evaluations that are based on this OHRQoL measure in India. Looking at the broader picture, up until recently patient morbidity has been interpreted through clinical measures like presence/absence of a particular symptom or disease. Oral health-related quality of life is particularly relevant in adolescence research, particularly when using the MID to interpret the differences between different socio-economic groups. This can be an essential step towards assessing oral health and evaluating oral health promotion interventions in those populations and settings.

Adolescents are in a critical stage of emotional, social and physiological development, which may make them potentially more susceptible to oral impacts. These impacts may affect their current quality of life and may well extend into adulthood. In the current study, a large proportion of the sample had oral impacts, particularly in terms of difficulties eating and brushing their teeth.

The study benefited from a high response rate (86.6%). The measures and questions used were adopted from internationally validated questionnaires and in turn were subsequently tested and validated among Indian adolescents. The study also went beyond the assessment of reliability and validity and provided an assessment of the MID for the Child-OIDP among the Indian adolescents.

In a large population based cross-sectional study, there is always a risk of reporting bias. Reporting bias may have arisen in this study in the form of a social responsiveness bias where adolescents might have given responses which according to them were socially desirable. Reporting bias could have also happened where adolescents well versed with the study might have given responses which they thought were wanted by the interviewer. Furthermore, the characteristics of this analytical sample, where all participants reported oral symptoms, may limit the applicability of the findings (both in terms of validity and also of MID) to the whole target population and therefore made them applicable to the section of the target population that reports oral health problems. Future studies should consist of samples that contain also adolescents that are free from oral symptoms.

Although measuring OHRQOL is challenging in adolescence due to developmental issues [39], the use of paediatric OHRQOL measures should be encouraged in order to gain insights into the assessment of impacts of oral conditions. Hence, reliable and valid relevant instruments are needed to facilitate collection of oral health-related quality of life data in adolescence. This study attempted to address some of the concerns regarding the lack of research on the use of oral health-related quality of life measures in this age group in India and has provided unique insight for adolescents living in extreme poverty. Further research should use patient reported outcomes and assess differences between clinical and perceived needs. Future studies should also complement the evaluation of the psychometric properties of the Child-OIDP in India by assessing its test–retest reliability, undertake analysis of its validity in different datasets and also collect longitudinal data to assess its responsiveness over time.

## Conclusion

Our analysis showed that the Child-OIDP is a reliable and valid OHRQoL measure among a socioeconomically diverse sample of 12–15 years old living in Delhi that included also adolescents living in extremely deprived settings.

## Data Availability

The datasets used and/or analysed during the current study are available from the corresponding author on reasonable request.

## Appendix

**Table:**

Oral symptom	n (%)
Toothache or sensitive teeth	546 (39.4)
Bleeding or swollen gums	387 (27.9)
broken tooth	263 (19.0)
Ulcer	321 (23.2)
Tooth discoloration	237 (17.1)

## References

[1] Wright J, Williams R, Wilkinson JR (1998). Health needs assessment: development and importance of health needs assessment. Br Med J.

[2] Sischo L, Broder HL (2011). Oral health-related quality of life: what, why, how, and future implications. J Dent Res.

[3] Castro RAL, Cortes MIS, Leão AT, Portela MC, Souza IPR, Tsakos G (2008). Child-OIDP index in Brazil: cross-cultural adaptation and validation. Health Qual Life Outcomes.

[4] Yusuf H, Gherunpong S, Sheiham A, Tsakos G (2006). Validation of an English version of the Child-OIDP index, an oral health-related quality of life measure for children. Health Qual Life Outcomes.

[5] Gherunpong S, Tsakos G, Sheiham A (2004). The prevalence and severity of oral impacts on daily performances in Thai primary school children. Health Qual Life Outcomes.

[6] Mtaya M, Astrøm AN, Tsakos G (2007). Applicability of an abbreviated version of the Child-OIDP inventory among primary schoolchildren in Tanzania. Health Qual Life Outcomes.

[7] Tubert-Jeannin S, Pegon-Machat E, Gremeau-Richard C, Lecuyer M-M, Tsakos G (2005). Validation of a French version of the Child-OIDP index. Eur J Oral Sci.

[8] Dorri M, Sheiham A, Tsakos G (2007). Validation of a Persian version of the OIDP index. No Title BMC Oral Health.

[9] Yusof ZY, Jaafar N (2012). A Malay version of the child oral impacts on daily performances (Child-OIDP) index: assessing validity and reliability. Health Qual Life Outcomes.

[10] Bernabé E, Sheiham A, Tsakos G (2008). A comprehensive evaluation of the validity of Child-OIDP: further evidence from Peru. Commun Dent Oral Epidemiol.

[11] Dhawan P, Singh A, Agarwal A, Aeran H (2019). Psychometric properties of Hindi version of child oral impact on daily performances (C-OIDP) index amongst school children in North India. J Oral Biol Craniofacial Res.

[12] Kumar S, Kumar A, Badiyani B, Kumar A, Basak D, Ismail MB. Oral health impact, dental caries experience, and associated factors in 12–15-year-old school children in India. Int J Adolesc Med Health. 2015; 2015(2). 10.1515/ijamh-2015-0041 26360493

[13] Guarnizo-Herreño CC, Watt RG, Fuller E, Steele JG, Shen J, Morris S (2014). Socioeconomic position and subjective oral health: findings for the adult population in England, Wales and Northern Ireland. BMC Public Health.

[14] Soe KK, Gelbier S, Robinson PG (2004). Reliability and validity of two oral health related quality of life measures in Myanmar adolescents. Commun Dent Health.

[15] Vettore MV, Aqeeli A (2016). The roles of contextual and individual social determinants of oral health-related quality of life in Brazilian adults. Qual Life Res.

[16] Tubert-Jeannin S, Pegon-Machat E, Gremeau-Richard C, Lecuyer M-M, Tsakos G (2005). Validation of a French version of the Child-OIDP index. Eur J Oral Sci.

[17] Montero-Martín J, Bravo-Pérez M, Albaladejo-Martínez A, Antonio Hernández-Martín L, María Rosel-Gallardo E. Validation the Oral Health Impact Profile Validation the Oral Health Impact Profile (OHIP-14sp) for adults in Spain. Vol. 1, J Clin Exp Dent. 2009. 19114956

[18] Agrawal N, Pushpanjali K, Gupta ND, Garg AK (2014). Child-Oral impacts on daily performances: A socio dental approach to assess prevalence and severity of oral impacts on daily performances in South Indian school children of Bangalore city: A cross-sectional survey. J Indian Assoc Public Health Dent.

[19] Kumar S, Debnath N, Ismail MB, Kumar A, Kumar A, Badiyani BK (2015). Prevalence and risk factors for oral potentially malignant disorders in Indian population. Adv Prev Med.

[20] Tsakos G, Allen PF, Steele JG, Locker D (2012). Interpreting oral health-related quality of life data. Commun Dent Oral Epidemiol.

[21] Schünemann HJ, Guyatt GH (2005). Goodbye (M)CID! Hello MID, where do you come from? (Commentary). Health Serv Res.

[22] Tsakos G, Bernabé E, D’Aiuto F, Pikhart H, Tonetti M, Sheiham A (2010). Assessing the minimally important difference in the oral impact on daily performances index in patients treated for periodontitis. J Clin Periodonto.

[23] Census of India Website: Office of the Registrar General and Census Commissioner, India. 2011.

[24] The Challenge of Slums: Global report on human settlements 2003. 2012.

[25] WHO. Oral health surveys; basic methods. 4th edn. Geneva: World Health Organization, 1997.

[26] Mathur MR, Tsakos G, Millett C, Arora M, Watt R (2014). Socioeconomic inequalities in dental caries and their determinants in adolescents in New Delhi, India. BMJ Open.

[27] The DHS Program-Wealth-Index-Construction. Available from: https://dhsprogram.com/topics/wealth-index/Wealth-Index-Construction.cfm

[28] Newton HJ, Baum CF, Beck N, Cameron AC, Epstein D, Hardin J (2010). The Stata Journal. Stata J.

[29] Gjersing L, Caplehorn JR, Clausen T (2010). Cross-cultural adaptation of research instruments: language, setting, time and statistical considerations. BMC Med Res Methodol.

[30] Åstrøm AN, Okullo I (2003). Validity and reliability of the oral impacts on daily performance (OIDP) frequency scale: a cross-sectional study of adolescents in Uganda. BMC Oral Health.

[31] Dorri M, Sheiham A, Tsakos G (2007). Validation of a Persian version of the OIDP index. BMC Oral Health.

[32] Gherunpong S, Tsakos G, Sheiham A (2004). Developing and evaluating an oral health-related quality of life index for children. The CHILD-OIDP Commun Dent Health.

[33] Streiner DL, Norman GR, Cairney J. Health measurement scales: a practical guide to their development and use.

[34] Mtaya M, Åstrøm AN, Tsakos G (2007). Applicability of an abbreviated version of the Child-OIDP inventory among primary schoolchildren in Tanzania. Health Qual Life Outcomes.

[35] Adulyanon S, Vourapukjaru J, Sheiham A (1996). Oral impacts affecting daily performance in a low dental disease Thai population. Commun Dent Oral Epidemiol.

[36] Masalu J, Åstrøm AN (2003). Applicability of an abbreviated version of the oral impacts on daily performances (OIDP) scale for use among Tanzanian students. Commun Dent Oral Epidemiol.

[37] Usha GV, Thippeswamy HM, Nagesh L (2013). Comparative assessment of validity and reliability of the Oral Impacts on Daily Performance (OIDP) frequency scale: a cross-sectional survey among adolescents in Davanagere city, Karnataka, India. Int J Dent Hyg.

[38] Pentapati KC, Acharya S, Bhat M, Rao SK, Singh S (2013). Oral health impact, dental caries, and oral health behaviors among the National Cadets Corps in South India. J Invest Clin Dent.

[39] Pal D (1996). Quality of life assessment in children; a review conceptual and methodological issues in multi-dimensional health status measures. J Epidemiol Commun Health.

